# The mastermind approach to CNS drug therapy: translational prediction of human brain distribution, target site kinetics, and therapeutic effects

**DOI:** 10.1186/2045-8118-10-12

**Published:** 2013-02-22

**Authors:** Elizabeth CM de Lange

**Affiliations:** 1 Division of Pharmacology, Leiden-Academic Center for Drug Research, Leiden University, Leiden, the Netherlands

**Keywords:** CNS drug therapy, Brain distribution, Translational research, Microdialysis, Target site concentrations, Prediction

## Abstract

Despite enormous advances in CNS research, CNS disorders remain the world’s leading cause of disability. This accounts for more hospitalizations and prolonged care than almost all other diseases combined, and indicates a high unmet need for good CNS drugs and drug therapies.

Following dosing, not only the chemical properties of the drug and blood–brain barrier (BBB) transport, but also many other processes will ultimately determine brain target site kinetics and consequently the CNS effects. The rate and extent of all these processes are regulated dynamically, and thus condition dependent. Therefore, heterogenious conditions such as species, gender, genetic background, tissue, age, diet, disease, drug treatment etc., result in considerable inter-individual and intra-individual variation, often encountered in CNS drug therapy.

For effective therapy, drugs should access the CNS “*at the right place*, *at the right time*, *and at the right concentration”*. To improve CNS therapies and drug development, details of inter-species and inter-condition variations are needed to enable target site pharmacokinetics and associated CNS effects to be translated between species and between disease states. Specifically, such studies need to include information about unbound drug concentrations which drive the effects. To date the only technique that can obtain unbound drug concentrations in brain is microdialysis. This (minimally) invasive technique cannot be readily applied to humans, and we need to rely on translational approaches to predict human brain distribution, target site kinetics, and therapeutic effects of CNS drugs.

In this review the term “Mastermind approach” is introduced, for strategic and systematic CNS drug research using advanced preclinical experimental designs and mathematical modeling. In this way, knowledge can be obtained about the contributions and variability of individual processes on the causal path between drug dosing and CNS effect in animals that can be translated to the human situation. On the basis of a few advanced preclinical microdialysis based investigations it will be shown that the “Mastermind approach” has a high potential for the prediction of human CNS drug effects.

## Introduction

Central nervous system (CNS) disorders are currently estimated to affect hundreds of millions of people worldwide [1]. While established treatments are currently available for most CNS disorders, significant unmet medical needs still remain. This is partly because currently available drugs merely treat symptoms rather than cure the disease, and may also elicit unwanted side effects. The attrition rate in CNS drug development is high and there is a need for revised approaches to improve CNS drug development and therapies.

It is often thought that the blood–brain barrier (BBB) hampers the adequate distribution of CNS drugs into the brain resulting in a lack of effects [2-4]. However, this cannot be the sole reason because other factors besides BBB transport determine the concentration-time profile (pharmacokinetics, PK) of the unbound drug at the brain target site [5]. Other important factors are plasma pharmacokinetics, plasma protein binding, cerebral blood flow, effective brain capillary surface area, blood-cerebrospinal fluid-barrier (BCSFB) transport, intracerebral distribution, CSF turnover, extracellular fluid (ECF) bulk flow, extra-intracellular exchange, brain tissue binding, and drug metabolism [5]. These factors are controlled by many processes, each of which has a specific influence [6], thereby playing a more or less important role in delivering the CNS drug to the *right place*, at the *right time*, and at the *right concentration*.

Apart from the multiple processes on the causal path between drug dosing and response, inter- and intra-individual variability in the contribution of each process to the ultimate CNS effect (pharmacodynamics, PD) need to be identified. This variability is caused by dissimilarities in genetic background, species, tissue, age, diet, disease, and drug treatment (heterogeneity) and associated differences in rate and extent of the individual processes on the causal chain between drug dosing and CNS effects. This explains why the same dose in different conditions may result in different CNS effects.

Investigations of the PK-PD relationship of a CNS drug should therefore be designed such that the contribution of a particular process is identified (for example by systematically influencing the process), and that information is obtained on time-dependency and on the unbound plasma and target tissue drug concentrations that drive the effect. To that end, advanced mathematical modeling is a prerequisite to learn about the contributions of individual processes in drug PK-PD relationships. This approach is here introduced as the “Mastermind approach”.

Noninvasive imaging techniques like positron emission tomography (PET), nuclear magnetic resonance (NMR) or (functional) magnetic resonance imaging ((f)MRI) are powerful methods to obtain information on transporter functionality [7,8], and target occupation [9,10]. These techniques may improve understanding of the influence of drug action on brain functionality in health and disease [11,12]. However, additional information is also needed about the unbound drug concentrations in the brain. In humans, at best, cerebrospinal fluid (CSF) concentrations can be obtained as a surrogate for brain target site concentrations [13-16], but the value of this surrogate is questionable [17]. To date, brain microdialysis is the only technique to obtain quantitative and time-resolution data on unbound extracellular drug concentrations in the brain (brain ECF) [18]. Although minimally invasive, microdialysis is a technique that can be applied in human brain only under highly restricted conditions [18-20]. Thus, we should pursue preclinical studies to learn about CNS target site distribution of drugs. This review will discuss the physiological factors involved in brain distribution and CNS effects, and the variability in these factors caused by heterogeneity. Furthermore it will provide examples of Mastermind approaches using microdialysis for quantitative assessment of 1) intracerebral distribution for drugs with different physico-chemical properties, 2) preclinical CNS target site concentrations following different routes of administration, 3) prediction of human CNS target site concentrations and CNS effects.

### Physiological factors in intracerebral distribution, drug target site kinetics, and CNS drug effects

**Figure 1:**
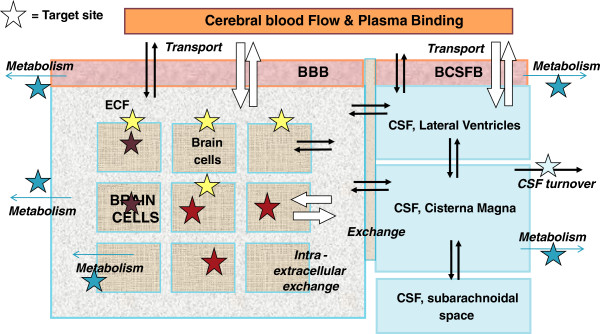
**Schematic presentation of the major compartments of the mammalian brain and routes for drug exchange; extracellular fluid (ECF), brain cells, lateral ventricular CSF, cisterna magna CSF and lumbar CSF, passive transport (black arrows) and active transport (white arrows), as well as metabolism and CSF turnover.** Drug targets may be present at different sites within the brain.

The anatomy of the CNS is complex and can grossly be divided into four main compartments [21-25]: the brain extracellular fluid (brain ECF) compartment, brain intracellular compartment, and the ventricular and lumbar CSF compartments. Transport of drugs into, within and out of the brain is governed by the blood–brain barriers, the anatomy of the brain parenchyma and fluid spaces, physiological processes, and drug-specific properties [26-32]. In combination, they determine the concentrations of a drug within a specific region of the CNS, including the unbound concentration at the target site that drives the effect (Figure 1). The players in drug exchange are briefly presented here.

#### Unbound concentrations in plasma

Only the unbound (free) drug is able to pass through membranes, and it is the unbound concentration in plasma that drives transport into the brain. Then, the unbound concentration at the CNS target site drives the interaction with the target and therewith the CNS effect (unbound drug hypothesis) [33-35]. In specific cases when the brain acts as a sink, total plasma concentrations may be relevant. Also, if a BBB transporter affinity and capacity is significantly larger than that for plasma proteins, “stripping” occurs and clearance can be based on the total plasma concentration. 

Unbound drug concentration is crucial for our understanding of drug transport and target interaction [36]. Often, the “unbound fraction” and “unbound concentration” are used interchangeably which leads to confusion: the “unbound fraction” is calculated from the ratio of unbound to total concentration [37-39]. So, it is the unbound concentration profile (kinetics) of the drug that should ultimately be taken into account to understand drug effects.

#### Transport across the brain barriers

**Figure 2:**
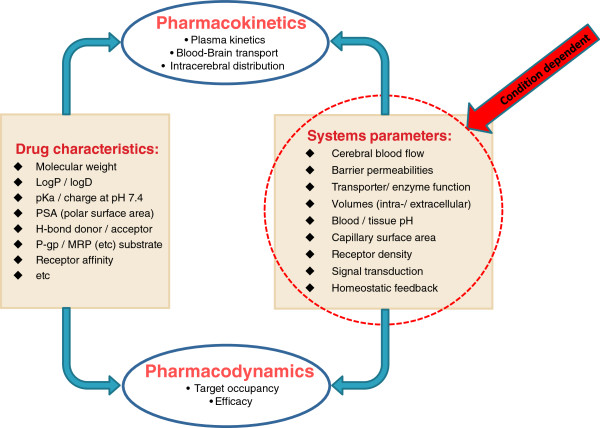
**Factors affecting the pharmacokinetics and pharmacodynamics of a drug.** The effects of a drug are determined on one hand by its physico-chemical/molecular characteristics and on the other hand by the properties of the biological systems involved.

The blood–brain barrier (BBB) and the blood-CSF-barrier (BCSFB) govern drug transfer into and out of the brain [40-44]. These barriers are comparable in many ways, but also have their specific characteristics [45-47]. The BBB consists of cerebrovascular endothelial cells while the BCSFB consists of choroid plexus epithelial cells. Together with the BBB and BCSFB transport characteristics and surface areas, the drug characteristics (lipophilicity, size, shape, charge, affinity for a transporter etc., Figure 2) determine the actual transport rate and extent. Recent investigations have indicated that the basal and apical membranes of the BCSFB have extensive infoldings and microvilli, respectively, suggesting that the BCSFB surface area, maybe the same order of magnitude as for the BBB [48].

There are a number of basic modes for compounds to move across brain barrier membranes [5,49,50]:

•Simple diffusion is a passive process driven by the concentration gradient, from high to low concentrations. The rate of diffusion is proportional to the concentration difference between compartments of the diffusing molecule. At equilibrium the concentration of the diffusing molecules are equal at both sides of the membrane. This mode of transport is size-dependent and permeability limited [51]. For hydrophilic drugs, not able to diffuse through lipophilic membranes, movement through the space between neighbouring barrier cells (paracellular transport) is restricted by the presence of tight junctions. [28,50].

•Facilitated diffusion is also a passive process from high to low concentrations but requires a helper molecule [52-54]. The rate of diffusion is limited by the availability of the helper molecules and at equilibrium the concentration of the diffusing molecules are equal on both sides of the membrane. Once all the helper molecules are saturated, increasing the concentration of diffusing molecules will only increase a waiting line for the helper molecules and will not increase rate of transport further. Facilitated transport is subject to competitive inhibition by substrate analogs and contributes to transport at the BBB of substances such as monocarboxyates, hexoses, amines, amino acids, nucleosides, glutathione, and small peptides.

•Fluid phase (vesicular) transport [55,56] includes bulk flow endocytosis (pinocytosis), adsorptive-mediated endocytosis, and receptor-mediated endocytosis [57,58]. Pinocytosis is the non-specific uptake of extracellular fluids. It is temperature and energy dependent, non-competitive, and non-saturable. Under physiological conditions, it occurs to a very limited degree in cerebral endothelial cells. Adsorptive-mediated endocytosis involves endocytosis in vesicles of charged substances by a non-specific mechanism [59,60]. Receptor-mediated transcytosis uses vesicles formed upon binding of large macromolecules to specific receptors [61]. At the BBB, transport of vesicles occurs only in direction from blood to brain. Vesicles may be subject to degradation within the cell, otherwise they are able to deliver their content to the abluminal side and into the brain.

•Active transport occurs by the action of membrane transport proteins for which transported molecules have a specific binding site. It requires energy and can transport substrates against a concentration gradient. Active transport is temperature sensitive and can become saturated. It can also be influenced by competitive and noncompetitive inhibitors and by interference with transporter protein phosphorylation by protein kinases. Transport proteins may have an important impact on drug development [62]. Transport systems [63] are directional (influx and/or efflux), and serve to maintain brain homeostasis for endogenous compounds. However, in numerous cases drugs may also be ligands for these transporters [64-70]. As a consequence, efflux transporters at the BBB have gained enormous attention over the last decade. Their presence accounts for the fact that many drugs, despite their lipophilic character favorable for passive transport, have a relatively poor brain distribution because they are substrates. The best known efflux transporters are P-glycoprotein (P-gp, or officially ABCB1 [71,72]), the multidrug resistance-related proteins (MRP’s, or officially ABCC’s [73]) and the breast cancer resistance protein (BCRP, or officially ABCG2 [74]), which all belong to the ABC transporter family [75].

#### Cerebral blood flow and effective capillary surface area

For drugs with high BBB permeation such that entry to the brain via the BBB capillaries is rapid, cerebral blood flow becomes rate-limiting. Cerebral blood flow can be influenced by changes in linear flow rate or by changes in the number of perfused capillaries. When the linear velocity of blood flow is increased, influx of highly permeable drugs across the BBB will increase (and *vice versa*), while BBB transport of slightly-to-virtually impermeable drugs will essentially be unchanged. Variations in the total number of the perfused capillaries in the brain (“effective perfusion”) will in theory affect BBB transport of all drugs [76,77].

#### CSF turnover and ECF bulk flow

CSF is produced by the choroid plexus [78] in the ventricles and leaves the CNS by re-absorption back into blood via the arachnoid villi in the subarachnoid space. CSF turnover [79] may reduce CSF drug concentrations [80]. The slower the permeation of a drug into the CSF, the more influence CSF turnover will have on the CSF concentration relative to its plasma concentration. Also, because of the relatively slow rate of CSF turnover in relation to trans-capillary transport, brain ECF concentrations will equilibrate more rapidly with plasma concentrations than with CSF. Furthermore, there is bulk flow of extracellular fluid into the CSF [42,81] that could counteract any molecular diffusion that might occur from the CSF into brain tissue through the ependymal linings of the ventricles [82].

#### Extra-intracellular exchange and brain tissue binding

Drugs may have their preference for extracellular or intracellular space, and may be subjected to nonspecific binding to brain tissue components [83]. Drug distribution between brain cells and extracellular space does not only occur by simple diffusion: active transport may also occur at brain cell membranes [68,84]. Distribution between extra- and intracellular compartments is very important for exposure of *unbound* drug concentrations at the target site (Figure 1) [85]. It can be seen that it is important to know the location of the target in order to optimize concentration profiles and drug effects.

#### Drug metabolism

Brain distribution may also be influenced by metabolism of the drug. This may occur at the level of the BBB and BCSFB, serving as “enzymatic barriers” to drug influx into brain, and also in the ependymal cells lining the CSF ventricles potentially influencing intracerebral distribution [86-89]. In brain blood vessels and closely-surrounding cell types, enzymes like cytochrome P450 haemoproteins, several cytochrome P450-dependent monooxygenases, NADPH-cytochrome P450 reductase, epoxide hydrolase, and also conjugating enzymes such as UDP-glucuronosyltransferase and α-class glutathione S-transferase have been detected. Several enzymes involved in hepatic drug metabolism have been found in brain microvessels and the choroid plexus. In the choroid plexus, very high activities (similar to those in the liver) have been found for UDP-glucuronosyltransferase and epoxide hydrolase, and several cytochrome P450 isoenzymes are also relatively high. Relatively high values of α and μ classes of glutathione S-transferase and glutathione peroxidase have been found in both the BBB and BCSFB.

#### Target interaction

The association and dissociation kinetics of a drug at the target (target interaction) is another factor to be taken into account for the relationship between drug concentration and CNS effect. Such interaction is not always instantaneous. For example, the opioid buprenorphine has slow kinetics for both receptor association and receptor dissociation. Such information was crucial to predict that reversal of respiratory depression caused by opioids could be achieved by the antagonist naloxone if naloxone is administered as a continuous infusion [90].

#### Signal transduction and homeostatic processes

It is frequently assumed that pharmacological responses depend solely on the extent of drug binding to its receptor (occupational theory). However, when observing tolerance, sensitization, dependence, and abstinence, it is clear that pharmacological responses *in vivo* can be subjected to modulation by homeostatic mechanisms. Thus, an integrative physiological approach is needed to understand concentration-effect relationships [91].

## Conclusion

Transport of drugs into the brain, within the brain and to the brain target site, and the resulting CNS effect are determined by many factors. Having information on just one of these factors in isolation is insufficient to predict target site distribution, let alone CNS drug effects.

### Heterogeneity as a source of variability in brain distribution and CNS effects

#### Heterogeneity

Mammals mostly share the same biological processes, which form the basis for interspecies extrapolation in drug development. However, problems arise with variable rates and extents in the processes on the causal path between drug administration and CNS effects. Below, examples of the impact of heterogeneity are addressed.

#### Genetic background

Genetic polymorphisms exist in the human MDR1 (P-gp) gene and may have clinical consequences [92,93]. In the clinical response to antidepressants, genetic factors in particular, are considered to contribute to variability. Variants affect the function of genes involved in both drug concentrations and CNS effects. Genetic variants affecting the metabolism of antidepressants may change pharmacokinetic factors, polymorphisms can affect receptor function, while signal transduction molecules may alter the pharmacodynamics [94]. A specific example is the effect of nicotine on heart rate. As much as 30% of the variance in the acceleration of heart rate was due to additive genetic sources, as determined in a study using a monozygotic and dizygotic twin population [95].

#### Species differences

Species differences occur in P-gp functionalities, also at the level of the BBB [7]. It was found that rhesus monkey P-gp is much closer to human P-gp than to beagle dog P-gp [96]. Also, the effects of inhibitors on P-gp functionality appear to be species dependent [97]. *In vivo* studies using PET imaging have also reported species differences in P-gp functionality [7].

#### Effect of gender

Sex hormones all influence the function and pathophysiology of the cerebral circulation [98]. Estrogen has numerous effects on dopamine neurotransmission, and because the incidence of Parkinson’s disease is lower in women than in men its possible use to either slow the progression or reduce the risk of Parkinson’s disease has been considered [99]. In schizophrenic patients, gender differences have been found in the pituitary secretion of prolactin, growth hormone, and thyroid-stimulating hormone in response to neuroleptic drug treatment [100]. Also, differences exist between female and male sensitivities to anesthesia and opioids [101].

#### Effect of age

Many studies indicate the importance of age in PK and/or PD. Age seems to affect P-gp functionality at the BBB [102], which may have consequences for brain efflux of P-gp substrates. Some of the properties of glucocorticoid receptors change with age [103]. Binding to the NMDA binding site by L-glutamate and/or antagonists, decreases with increasing age in the cerebral cortex and hippocampus, regions that are important for memory processing [104]. Important changes starting at mid-life in neuroanatomy, neurochemistry and endogenous pain inhibition may be associated with alterations in pain sensitivity [105]. Another example is impaired neurotransmission that may be responsible for at least some of the behavioral abnormalities associated with aging [106].

#### Effect of diet

Mulder *et al.*[107] have shown that the *combination* of a high-fat diet and APOe4 knockout conditions in mice resulted in a loss of BBB functionality. This leads to an increase BBB permeability, resulting in increased IgG staining and increased fluorescein distribution in the brain. Also, red wine polyphenolic contents influence Alzheimer’s disease-type neuropathology and cognitive deterioration, in a component-specific manner [108].

#### Disease states

In the rat pilocarpine model of epilepsy, increased brain concentration of the active metabolite of oxcarbazepine was observed following seizures together with inhibition of BBB efflux transport, but without changes in plasma concentrations. This indicated that a distributional process is changed at the level of the BBB in epileptic conditions [109]. Changes in BBB permeability during electrically-induced seizures in human have also been observed [110]. A change in P-gp expression at the BBB has been reported in humans with the human immunodeficiency virus [111]. Tunblad *et al.* reported the impact of meningitis on morphine distribution in piglet brain, indicating decreased BBB functionality [112]. Also, after subcutaneous infusion of rotenone in rats, changes in BBB permeability for fluorescein occur as a result of induced peripheral inflammation but without any biomarkers for Parkinson’s disease [113]. In contrast, the unilateral brain infusion of rotenone did induce biomarkers for Parkinson’s disease, but no changes in BBB permeability for fluorescein and the large neutral amino acid transporter-mediated BBB transport of L-DOPA [114].

#### Drug treatment

Cleton *et al.*[115] found changes in the relationship between long-term treatment effects of midazolam and its concentration-EEG effect which, however, were unrelated to changes in benzodiazepine receptor function. Other examples are the alterations in striatal neuropeptide mRNA produced by repeated administration of L-DOPA, ropinirole or bromocriptine which appeared to correlate with dyskinesia induction in MPTP-treated marmosets [116], the tolerance to diazepam after chronic use [117], and the onset of hyperalgesia by opioid treatment [118].

#### Heterogeneity results in variability

Heterogeneity in genetic background, species, gender, tissue, age, diet, (pathologic) conditions, drug treatment, are underlying the variability in rate and extent of individual processes. This explains why the same dose in different subjects may result in different effects. It is therefore surprising that, in most cases, the dose-effect or at best the plasma-effect relationships continue to be used for extrapolation.

### Need for quantitative and integral [“mastermind”] approaches

#### Heterogeneity

As has been shown, there are many factors that play a role in the PK-PD relationships of CNS drugs. The rates and extents of the multiple processes on the causal path between drug dosing and CNS can be highly diverse. Therefore, data obtained in a particular condition are not necessarily predictive of that in another condition. But, as living mammals mostly share the same biological processes, knowledge of rate and extent of individual processes provide the foundation for interspecies extrapolation in drug development [119-122].

#### Translation from animal to human, the mastermind approach

Because in the body (biological system) multiple processes as are working concurrently, there is a need for integrated *in vivo* experiments. This means that the experiments should obtain data on multiple processes as much as possible from the same subject, in a time-dependent and quantitative manner. This also means that we have to address heterogeneity of the rates and extents of physiological processes on the causal path between drug administration and CNS effects and have to use study designs in which individual processes can be challenged. This can be done, for example, by changing plasma protein binding [123,124], inhibition of a particular efflux transporter [125], blocking particular receptors [126,127], or by induction of a pathological state [113,128] and enabling us to learn about the contribution of individual processes in CNS target site kinetics [17] and dynamics [129,130].

Here is the place to introduce the term “Mastermind approach” as an allegory. In the game “Mastermind” there are pins with different colors, and different positions in which part of the colors can be positioned. By systematically and strategically varying the position and colors of the pins the “code” can be ultimately deciphered. With each colors representing a particular mechanism, the code represents a particular PK-PD relationship. Of course, the dose-effect relationship of CNS drugs includes many more variables than the number of differently colored pins in the Mastermind game, and this is the reason that we just cannot interpret the data solely by “eye-ball analysis” and need to use advanced mathematical modeling [30,31,129-132]. In doing so, we need to make a strict distinction between the properties of drugs and the properties of biological systems to predict drug behavior under different conditions.

The physiologically-based pharmacokinetic (PBPK) modeling approach has provided the basis for interspecies extrapolation, has focused on quantitative modeling of mass transport into and out of physiological compartments, and has made highly significant contributions to knowledge of systems and the fates of drugs [133]. It has not, however, specifically taken into account the distinction between the bound and unbound drug. With the introduction of the microdialysis technique, information on unbound drug concentrations has become available and is providing the next step in physiologically-based modeling. Below, studies are presented that explicitly show the value of knowledge of unbound drug concentrations, as obtained by intracerebral microdialysis.

### Applications of the mastermind approach

#### Impact of drug properties on intracerebral distribution

**Figure 3:**
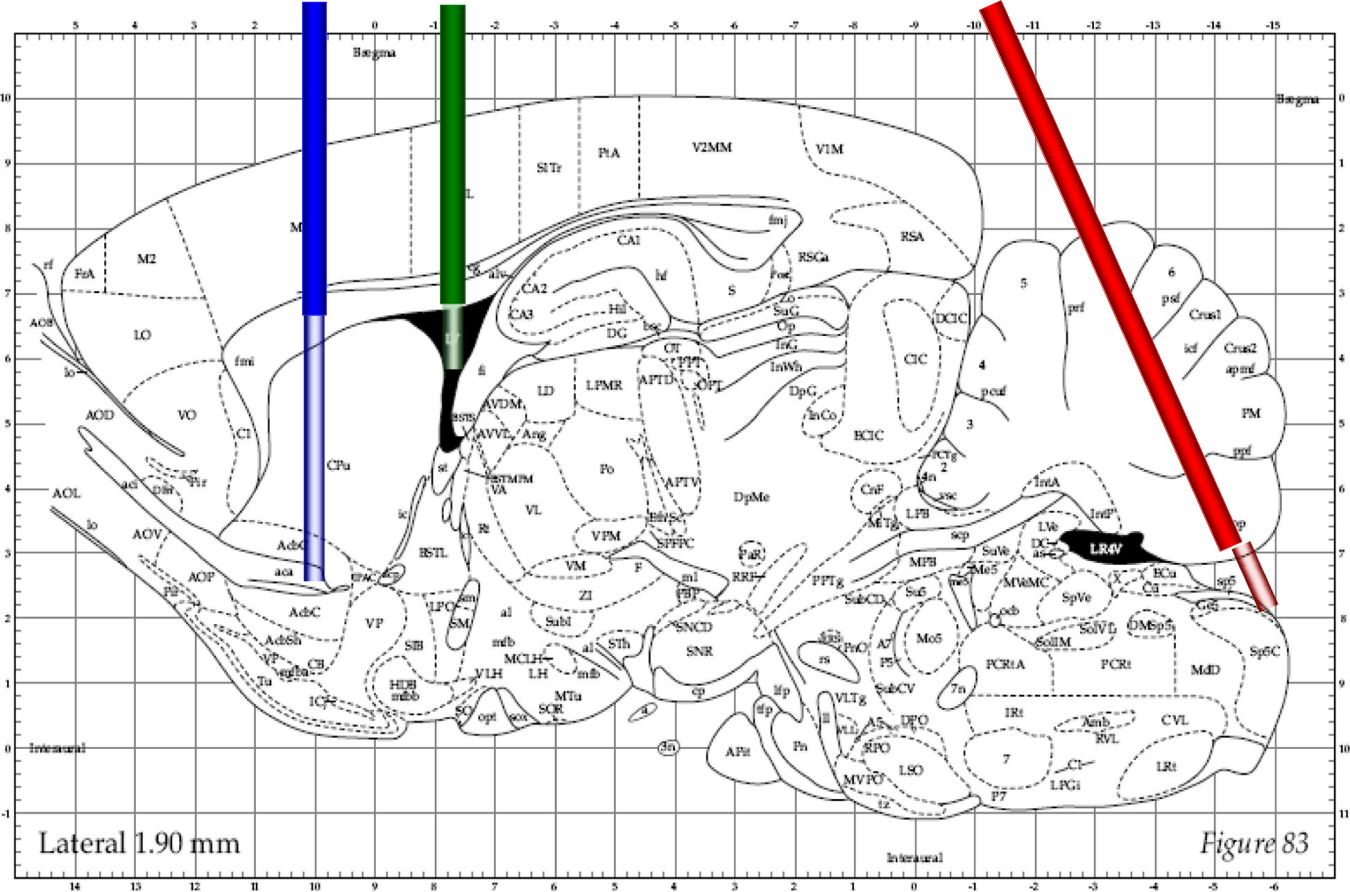
**Longitudinal section of the rat brain (From: Paxinos and Watson **[136]**) with the positions of the microdialysis probes indicated. From left to right: probe position in striatum, lateral ventricle CSF and cisterna magna CSF, respectively.**

For prediction of CNS drug action, it is important to have information of unbound drug concentrations at its CNS target site in humans. However, this is limited by the inaccessibility of the human brain for sampling. Moreover, it is often difficult to quantify human CNS drug effects indicating that effects in humans should be predicted by other approaches. As a surrogate for the concentrations of unbound drug at target sites, CSF concentrations are often used and considered appropriate [16,83], however, a generally applicable relationship between CSF and brain ECF concentrations is questionable [5,15,17,134]. Therefore, it is of interest to investigate the relationship between the two, for different drugs and under different conditions, to discover what general principles exist. In our laboratory such studies were performed for acetaminophen [135] and quinidine whose physico-chemical properties are shown in Table 1. Experiments in rats were performed using intravenous drug administration and concurrent sampling of blood and collection of microdialysis fluid from probes located in brain striatum ECF, lateral ventricle CSF, and cisterna magna CSF (Figure 3).

•Acetaminophen:

**Table 1:** Physico-chemical properties of acetaminophen and quinidine

**Compound**	**MW**	**PSA**	**logP**	**logD[7.4]**	**pKa1 [Acid]**	**pKa2 [Acid]**	**pKa1 [Base]**	**pKa1 [Base]**	**Ionized at physiological pH**	**Substrate for**
Acetaminophen	151	49,3	0,25	0,23				10,2	0% [neutral]	-
Quinidine	324	45,6	2,29	1,4	4,2		8		99.8% [positive]	Pgp

MW = molecular weight, PSA = polar surface area, Log P = measure of lipophilicity determined as log of partition of un-ionised compound over octanol/water, Log D[7.4] = measure of lipophilicity at physiological pH, determined as log of distribution of the compound over octanol/ buffer pH = 7.4.

**Figure 4:**
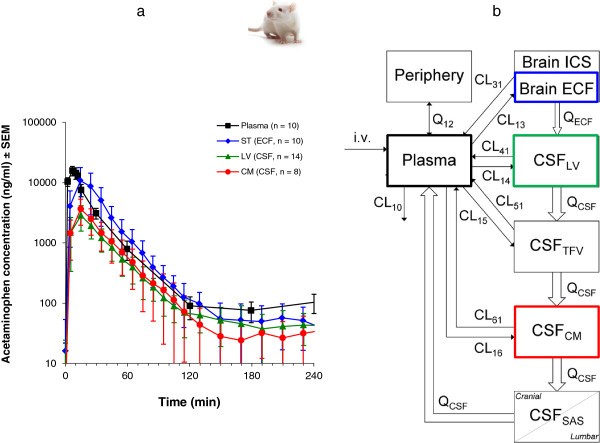
**Brain distribution of acetaminophen in the rat. a**) Data obtained for acetaminophen in the rat following an intravenous dose of 15 mg/kg, administered by constant-rate infusion for 10 minutes. The data are presented as the average (geometric mean ± SEM) of the observed unbound acetaminophen concentration-time profiles in plasma (black, n = 10), striatum ECF (ST, blue, n = 10), CSF in lateral ventricle (LV, green, n = 14), and CSF in cisterna magna (CM, red, n = 8). The data show that brain ECF (striatum) concentrations are comparable to those in plasma and significantly higher than those in both the lateral ventricle and the cisterna magna CSF compartments. **b**) The physiologically-based pharmacokinetic model for the rat developed on the basis of the data obtained for acetaminophen as shown in a). This model describes the obtained data adequately, and predicts the CSF acetaminophen concentrations in the third and fourth ventricle (lumped as TFV) as well as in the subarachnoid space (SAS), the latter being most representative of the lumbar CSF concentrations [135]. Denotations: In the model clearance (CL, volume/time), and ECF bulk or CSF flow (Q, volume/time) are indicated. Numbering indicates exchange between different compartments: 12 from plasma to peripheral compartment; 21 from peripheral to plasma compartment; 13 from plasma to brain ECF compartment; 31 from brain ECF to plasma compartment; 14 from plasma to CSF_LV_ compartment; 41 from CSF_LV_ to plasma compartment; 15 from plasma to CSF_TFV_ compartment; 51 from CSF_TFV_ to plasma compartment; 16 from plasma to CSF_CM_ compartment; and 61 from CSF_CM_ to plasma compartment.

**Figure 5:**
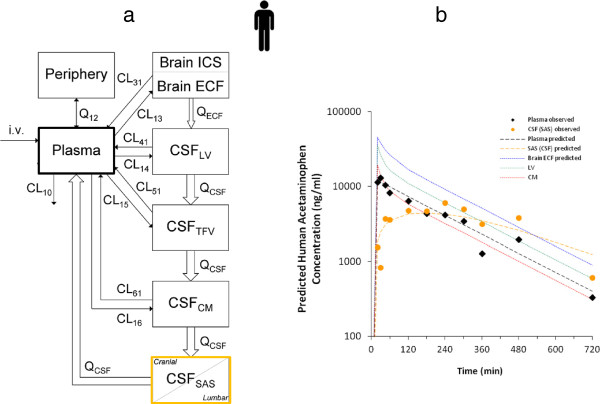
**Observed and predicted distribution of acetaminophen in human brain. a**) The human physiologically-based pharmacokinetic model which equals the rat physiologically-based pharmacokinetic model, but includes human instead of rat physiological parameters. (For the denotations in the model see Figure 4b). **b**) Acetaminophen concentrations in human plasma and brain. Data points represent observed data in human for plasma (black diamonds) and lumbar CSF (orange circles) by Bannwarth *et al.*[137]. Lines represent predictions of human plasma concentrations (black line), human lumbar CSF concentrations (orange line, and human brain ECF concentrations (blue line) by the “humanized” preclinical physiologically-based PK model [135].

For acetaminophen the resulting unbound concentration-time profiles in plasma, brain ECF and CSF in lateral ventricle and cisterna magna are presented in Figure 4[135], and indicate rapid equilibration with plasma concentration. However, brain ECF concentrations are on average 4-fold higher than CSF concentrations, with average brain-to-plasma [AUC_0-240_] ratios of 1.2, 0.30 and 0.35 for brain ECF, lateral ventricle CSF and cisterna magna CSF, respectively. This shows that even for a compound with only passive transport into, within and out of the brain, differences exist between brain ECF and the CSF pharmacokinetics. A physiologically-based pharmacokinetic model was developed [135]. This model included the central (plasma) and peripheral tissue compartments and, for the brain, the brain intracellular space (brain ICS), brain extracellular fluid (brain ECF), lateral ventricle CSF, cisterna magna CSF and also subarachnoid space CSF (CSF SAS) was included. The latter is important with regard to prediction of lumbar CSF concentrations in human, as lumbar CSF is part of the SAS CSF that can be distinctively different from ventricular or cisterna magna CSF (as predicted for acetaminophen by this model [135]). This physiologically-based pharmacokinetic model was turned into a human model, by replacing the rat physiological parameters by those in human (Table 2). The resulting model was used to predict plasma and CSF concentrations in human, and the plasma and SAS CSF concentrations of acetaminophen predicted by the model could be compared to actual data obtained in human by Bannwarth *et al.*[137]. The model successfully predicted the available human plasma and SAS CSF data (Figure 5). This gives us confidence in the method for prediction of human brain ECF concentrations, as best possible reflection of target site concentrations.

•Quinidine:

**Table 2:** Values of rat and human physiological parameters

**Physiological parameter**	**Rat value**	**Human value**
Brain ECF volume	290 μl	240 ml
Total CSF volume	300 μl	140 ml
Brain ECF flow	0.2 μl/min	0.2 ml/min
CSF flow	2.2 μl/min	0.4 ml/min
Lateral ventricle volume	50 μl	25 ml
Cisterna magna volume	17 μl	7.5 ml
Subarachnoid space volume	180 μl	90 ml

**Figure 6:**
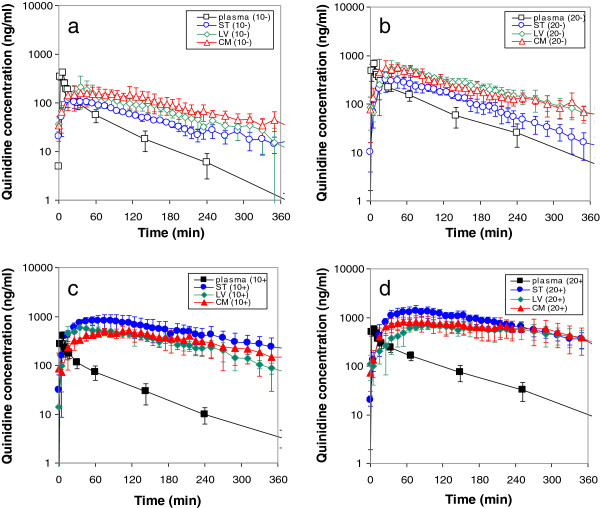
**Brain distribution of quinidine in the rat [Westerhout J, Smeets J, Danhof M, De Lange ECM: The impact of P-gp functionality on non-steady state relationships between CSF and brain extracellular fluid. J Pharmacokin Pharmacodyn, submitted]**. Average (geometric mean ±SEM) unbound quinidine concentration-time profiles following: **a**) 10 mg/kg, with co-administration of vehicle (-); **b**) 20 mg/kg, with co-administration of vehicle (-); **c**) 10 mg/kg with co-administration of 15 mg/kg tariquidar (+), and **d**) 20 mg/kg with co-administration of 15 mg/kg tariquidar (+). Black, blue, green and red symbols represent plasma, brain ECF, lateral ventricle CSF and cisterna magna CSF, respectively. Open symbols indicate data obtained without (-) and closed symbols represent data obtained with (+) the P-gp blocker tariquidar, respectively. The data show substantially *lower* concentrations in brain ECF (striatum) compared to lateral ventricle and cisterna magna CSF concentrations for both the 10 and 20 mg/kg dose (**a**, **b**). Upon co-administration of tariquidar, the brain ECF (striatum) concentrations were *higher* than those in the CSF compartments (**c**, **d**). These data show that the relationship between brain ECF and CSF concentrations is influenced by P-gp-mediated transport.

The same experimental setup was used for quinidine, a paradigm lipophilic compound and P-gp substrate. To investigate the specific contribution of P-gp-mediated transport, quinidine was administered at two different intravenous dosages, both with and without co-administration of tariquidar as P-gp transport inhibitor [Westerhout J, Smeets J, Danhof M, De Lange ECM: The impact of P-gp functionality on non-steady state relationships between CSF and brain extracellular fluid. J Pharmacokin Pharmacodyn, submitted]. Figure 6 shows the resulting kinetics of unbound quinidine in plasma, brain ECF, lateral ventricle CSF and cisterna magna CSF. Apart from the unexpected finding that brain ECF concentrations of quinidine were higher than the unbound quinidine concentrations in plasma (indicating an active influx that has not been identified before), substantial *lower* concentrations in brain ECF (striatum) compared to lateral ventricle and cisterna magna CSF were found for both the 10 and 20 mg/kg dose (Figure 6 a,b). Upon co-administration of tariquidar, plasma concentrations remained similar, while brain concentrations for all compartments were substantially increased. Interestingly, now the brain ECF (striatum) concentrations were *higher* than those in the CSF compartments (Figure 6 c,d). These data clearly show that the relationship between brain ECF and CSF concentrations is influenced by P-gp-mediated transport. It underscores the importance for more mechanistic insights into the processes that govern CNS drug concentrations at different sites in the brain.

#### Impact of route of administration on brain target site kinetics and CNS effects

The effects of therapeutic agents following oral administration are often limited due to active first-pass clearance by the liver and restricted BBB transport. Apart from rapid uptake of compounds from the systemic circulation, intranasal administration may provide the only direct route for non-invasive delivery of therapeutics into the CNS [138-140]. Intranasal administration could enhance the CNS target site bioavailability and therewith provide a more selective effect of CNS drugs [49,141,142]. However, the immediate need is for quantitative information on both the rate and extent of delivery in relation to the action of nasally-administered drugs.

**Figure 7:**
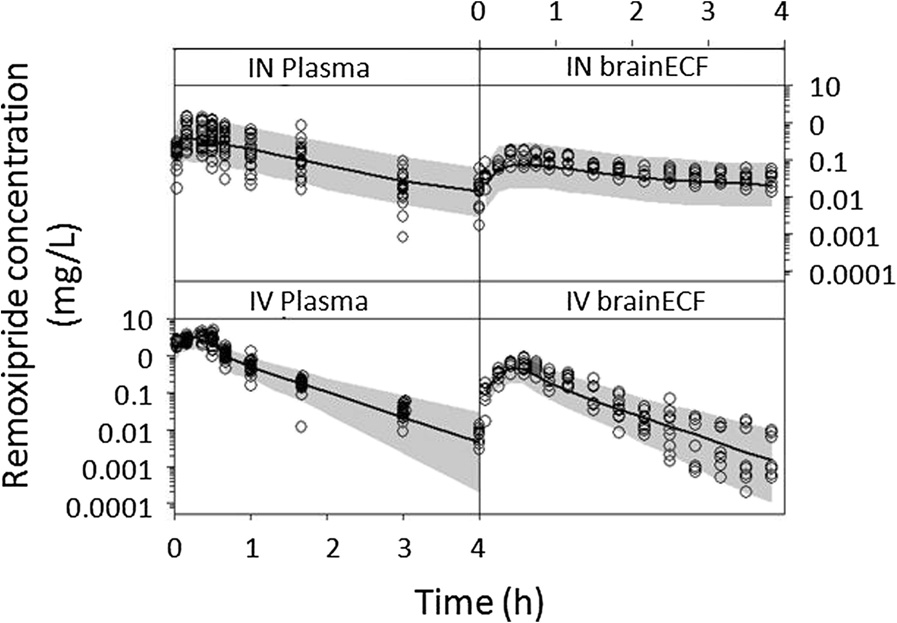
**Brain distribution of remoxipride (REM) in the rat following intravenous (IV) and intranasal (IN) administration.** Observed data points for plasma and brain ECF concentrations in the rat following intranasal and intravenous administration of remoxipride (open circles), and the “visual predictive check (VPC)” of the median concentration predictions of the model (black line), and the 90% prediction intervals (grey area). The VPC indicated that the model adequately described the observed data (from [147] with permission).

•Advanced mathematical PK model on remoxipride distribution in brain:

The recently-developed minimum-stress and freely-moving rat model for intranasal drug administration [143], was used together with serial sampling of plasma and brain microdialysate. The dopamine D2 receptor antagonist, remoxipride, was administered at three different doses via the intranasal or intravenous route. An advanced pharmacokinetic model was developed using the data obtained after intravenous dosing. For good prediction of the intranasal data, the model had to be extended with two absorption compartments, one for absorption from the nose into the systemic circulation, and one for absorption from the nose direct to the brain. The final model gave a good prediction on all observed data [144]. Figure 7 shows the actual observed data points for plasma and brain ECF concentrations in the rat following intranasal and intravenous administration of remoxipride (open circles). In addition, in Figure 7 the results of the so called “visual predictive check (VPC)” are displayed, as the prediction of the median concentration predictions of the model (black line), and the 90% prediction intervals (grey area). The VPC indicated that the model well described the observed data.

The absorption process could be described in terms of rates and extent (bioavailability). About 75% of the intranasal dose was directly absorbed into the brain. Unexpectedly, the direct nose-to-brain absorption did not turn out to be a rapid route *per se*. For remoxipride, the rate was slow, explaining prolonged brain ECF exposure after intranasal compared to intravenous administration. This is the first time that both rate and extent of delivery have been identified quantitatively and is of utmost importance for optimizing direct nose-to-brain delivery, by varying drug properties and formulation [144].

•Advanced mathematical PK-PD model on remoxipride brain distribution and effects:

The advanced pharmacokinetic model on remoxipride brain distribution following intranasal and intravenous dosing was further developed to a PK-PD model. To that end, the plasma levels of the pituitary hormone prolactin, obtained in the same rats, were used as a biomarker of D2 receptor antagonism [145-147]. Furthermore, baseline variations in plasma prolactin concentrations were investigated [148]. Also, the prolactin response was measured following double low dosing of remoxipride at different time intervals to get information on the synthesis of prolactin in the pituitary lactotrophs [149,150]. The final PK-PD model consisted of 1) a pharmacokinetic model for plasma and unbound brain remoxipride concentrations, 2) a pool model for prolactin synthesis and storage, and its release into- and elimination from plasma, 3) a positive feedback of prolactin plasma concentrations on prolactin synthesis, and 4) the brain unbound concentrations of remoxipride for the inhibition of the D2 receptor, and resulting stimulation of prolactin release into plasma.

In conclusion, this mastermind approach allowed the explicit separation and quantitation of systemic and direct nose-to-brain transport after intranasal administration of remoxipride in the rat, and showed that the brain unbound concentrations could be directly linked to the effect. The model included parameters for the underlying processes of synthesis, storage and release of the pituitary hormone, and the positive feedback of its synthesis by prolactin plasma levels. The latter was in contradiction to a previous report [148]. An important finding was that indeed the brain unbound remoxipride concentrations were indistinguishable from target site concentrations to drive the release of prolactin into plasma. Such mechanistic information should be useful to extrapolate/predict the effects of remoxipride in humans.

#### Prediction of human target site kinetics and associated drug effects

Quantification of drug- and biological system specific parameters in translational mathematical models provides the opportunity to re-scale the animal model up to humans [129-131,151-153]. Allometric scaling of drug pharmacokinetic properties and the biological system-specific parameters have been used in previous translational investigations to predict drug effects in humans with a reasonable degree of success, [154,155]. Compared to pharmacokinetic properties, pharmacodynamic properties are more difficult to scale [156], since they are not often related to bodyweight (e.g. receptor occupancy, transduction, maximal effect, etc.). However, this information can be obtained from *in vitro* bioassays [157]. For many drugs and endogenous compounds, clinical information is readily available in literature [158-161]. This provides the opportunity to replace rat biological system parameters by human values, and to provide an extrapolation step from rat to human. At an early stage in drug development, such information can be used for simulation and to provide preliminary insight on the clinical applicability of a drug.

**Figure 8:**
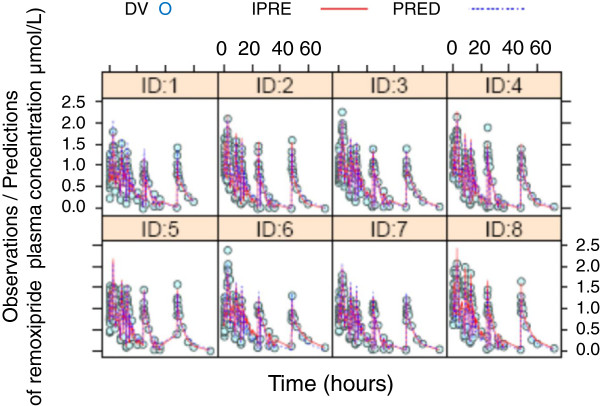
**Observed and model prediction of remoxipride concentrations in human plasma (from** [148,151,165] **with permission).** Human data on remoxipride and prolactin plasma concentrations were obtained following double intravenous administration of remoxipride at different time intervals. Data points on remoxipride concentrations in plasma (y-axis, concentration of remoxipride in μmol/L) as a function of time (x-axis, time in hours) are presented for each individual human subject (open symbols, DV). Using allometric scaling the preclinical PK model of remoxipride was tuned to the human PK model. The human PK model successfully predicted the remoxipride plasma kinetics in humans: the individual prediction of the median remoxipride concentrations is indicated (IPRE, ^________^) as well as the population prediction (PRED, ---------).

To test the predictive value of the preclinical PK-PD model of remoxipride [144,147], allometric scaling and literature data [162] were used to tune the preclinical PK-PD model, from rat systems to that of human [147]. Human data on remoxipride and prolactin plasma concentrations were used, being obtained following double intravenous administration of remoxipride at different time intervals [149]. The translational PK-PD model successfully predicted the remoxipride plasma kinetics in humans (Figure 8) as well as system prolactin response in humans, indicating that positive feedback on prolactin synthesis and allometric scaling thereof could be a new feature in describing complex homeostatic processes [147].

## Conclusions

Drug properties and biological system properties together determine intracerebral distribution of drugs and subsequent CNS effects. The fact that rate as well as extent of the biological processes are dynamically regulated and therefore may be condition dependent, explains the high intra and inter-individual variability encountered in CNS drug effects. We also need to understand the sources of variability in CNS drug effects to be able to improve drug development and therapies. Moreover, as these processes are working concurrently, and together determine the final CNS effect, they cannot only be studied in isolation, indicating the need for integrated *in vivo* experiments.

In these experiments data on plasma PK, brain distribution and CNS effects of a drug should be obtained from the same setting as much as possible. In addition, time-dependency should be explicitly included, and information should be obtained on the unbound drug. Then, the contribution of a certain process in the PK-PD relationship can be deduced, either by changing experimental conditions in a controlled manner (e.g. blocking of an active transport process, or irreversible binding of part of particular receptors), or by performing the same experiment for a different drug, and the use of advanced mathematical modeling. This approach is here introduced as the “Mastermind approach”. Examples given of this approach show that data from preclinical translational models in principle are able to predict human CNS drug distribution, target site kinetics, and CNS drug effects.

## Abbreviations

BBB: Blood–brain barrier;BCSFB: Blood-CSF-barrier;Brain ECF: Brain extracellular fluid;CNS: Central nervous system;CSF: Cerebrospinal fluid;ECF: Extracellular fluid;P-gp: P-glycoprotein

## Competing interests

The author declare that She have no Competing Interest

## Author’s contribution

EL had the overall supervision on the data generation and modelling, and performed the writing of the manuscript.
